# Editorial: Human milk, nutrition and infant development, volume II

**DOI:** 10.3389/fnut.2026.1812756

**Published:** 2026-03-17

**Authors:** Francisco J. Pérez-Cano, Veronique Demers-Mathieu, Claude Billeaud

**Affiliations:** 1 Physiology Section, Department of Biochemistry and Physiology, Faculty of Pharmacy and Food Science, University of Barcelona (UB), Barcelona, Spain; 2 Institute of Research in Nutrition and Food Safety (INSA), UB, Santa Coloma de Gramenet, Spain; 3 Janssen Pharmaceutical Companies of Johnson & Johnson, San Diego, CA, United States; 4 Centre Hospitalier Universitaire de Bordeaux, Bordeaux, France; 5 European Association for Paediatrics Education (AEEP/EAPE), Bordeaux, France

**Keywords:** breastfeeding, breast milk, epigenetic, immunoglobulins, microbiota, oligosaccharides, PUFA

**Figure 1:**
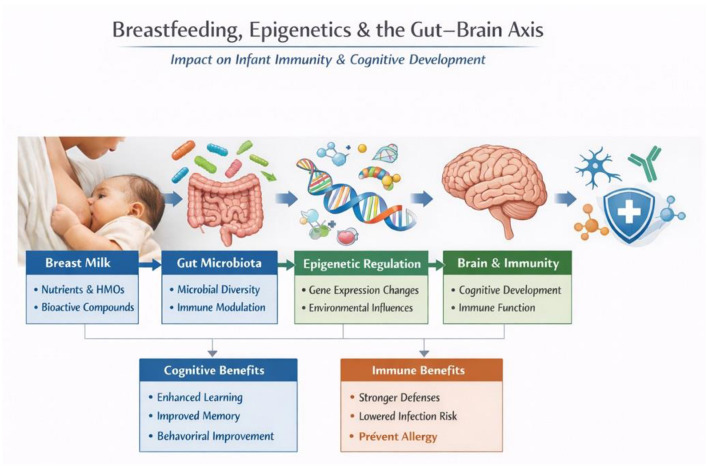
Overview of the benefits of breast milk. 1. Breast Milk provides essential nutrients, human milk oligosaccharides (HMOs), and bioactive compounds crucial for infant development. 2. Microbiota: Promotes microbial diversity and supports immune modulation. 3. Epigenetic Regulation: Influences gene expression changes and adapts to environmental factors. 4. Brain & Immunity: Enhances cognitive development and strengthens immune function. 5. Benefits: Cognitive benefits include improving learning and memory, while immune benefits include stronger defenses, reduced infection risk, and allergy prevention.

Human milk (HM) is universally recognized as the gold standard for infant nutrition, immunity, and neurodevelopment. Its biologically complex and dynamic composition is uniquely tailored to meet the evolving needs of infants, providing a sophisticated blend of macronutrients, bioactive compounds, and immunological factors that far surpass the capabilities of formula (Figure 1). The multifaceted benefits of HM extend beyond immediate nourishment, playing a pivotal role in epigenetic programming and shaping long-term health and cognitive outcomes.

The comprehensive body of research reviewed herein—compendium of 19 up-to-date original research and review papers -underscores the critical importance of HM in supporting infant health and development. Specifically, this Research Topic compile articles focused on the impact of HM aspects related to immunity, programming and infection, neurological and growth infant development but also focused on innovations and challenges.

Regarding growth, HM delivers optimal nutrition through a distinctive composition of macronutrients, micronutrients, and bioactive elements. HM proteins, such as lactoferrin and α-lactalbumin, are fundamental for growth, digestion, and immune function, which, offer an unique bioactivity that is not replicated in infant formula (Chauvet et al.). Long-chain polyunsaturated fatty acids (LCPUFAs), including docosahexaenoic acid (DHA) and arachidonic acid (ARA), are essential for brain and retinal development, directly influencing cognitive, language, and motor skills (Turner et al.). Notably, DHA concentrations in HM are positively correlated with enhanced cognitive outcomes (Turner et al.). Nervonic acid (NA), present in HM, is vital for brain myelination and cognitive function, especially in preterm infants, with its highest levels found in colostrum and diminishing as lactation progresses (Destaillats et al.). The fortification of donor milk, particularly for very low birth weight infants, has been shown to improve growth metrics and reduce complications, such as necrotizing enterocolitis, with individualized fortification methods yielding superior outcomes (Ong et al.).

HM serves also as a robust immunological shield for the infant, conferring both passive and active immunity. Human milk oligosaccharides (HMOs) act as prebiotic sugars that foster the proliferation of beneficial gut bacteria, such as *Bifidobacterium*, and function as decoy receptors to inhibit pathogen adhesion and modulate immune responses (Slater et al.). HMOs are instrumental in reducing inflammation and promoting immune tolerance, while also protecting against viral infections and allergic asthma by encouraging regulatory immune responses (Rijks et al.). In addition, circadian variations in milk components, including melatonin and cortisol, play a role in regulating infant sleep-wake cycles and supporting immune system maturation (Woortman et al.). Finally, HM microbiome, shaped by maternal factors, delivery mode, and infant age, is crucial in establishing the infant gut microbiota and immune system (Sun et al.). Altogether, HMOs influence the development of the gut microbiome by promoting beneficial bacteria to produce short-chain fatty acids (SCFAs), which are integral to brain energy metabolism, inflammation control, and neurotransmitter synthesis (Wang et al.).

In addition, HM interacts with infant saliva and enhances mucosal immunity and antioxidant protection. HM bioactive compounds, including cytokines, growth factors, immune proteins and cells, safeguard mucosal surfaces and mitigate oxidative stress, a function particularly critical for preterm infants with underdeveloped immune systems (Song and Kim). The combined action of salivary antioxidants and HM components contributes to the maturation of mucosal immunity, lowering the risk of infections and inflammation in the oral cavity and gastrointestinal tract (Song and Kim).

HM not only acts on immune development;, but also has a role in brain development. HM is uniquely equipped to foster and build infant cognitive and neurological foundations, laying the groundwork for lifelong brain health. Fatty acids, such as DHA and ARA, are associated with improved cognitive, language, and motor development, with outcomes influenced by maternal secretor status and infant sex (Turner et al.). Nervonic acid (NA) supports brain myelination and cognitive function, particularly in preterm infants (Destaillats et al.). Supplementation of infant formula with milk fat globule membrane (MFGM) has been shown to enhance brain development and cognitive abilities, especially in non-verbal and fine motor domains (Zhou et al). The gut-brain axis, a bidirectional communication network between the gut microbiota and the brain, is enhanced by HMOs. HMOs promote beneficial bacteria producing SCFAs, which cross the blood-brain barrier and influence brain energy metabolism and neurotransmitter synthesis (Amin et al.). Moreover, circadian rhythms in HM components provide essential cues for infant sleep-wake cycles and overall development (Woortman et al.).

Epigenetic programming can be HM dependent, as HM can influence gene expression and long-term health trajectories. Indeed, exosomal microRNAs present in HM regulate Wnt signaling gene expression that promotes β-cell proliferation and maturation, which helps to reduce the risk of type 2 diabetes and other metabolic disorders (Melnik et al.). HMOs and sialic acid further support neurodevelopment through epigenetic mechanisms, facilitating brain ganglioside development and cell signaling (Turner et al.).

Despite its unparalleled benefits, breastfeeding is not without obstacles and some challenges should be considered. Pain during breastfeeding can impede mothers from providing optimal nutrition and immunity. However, osteopathic manipulative treatments alleviated pain and improved breastfeeding rates (Elleau et al.). Maternal stress, which elevates cortisol levels in breast milk, may adversely affect infant health and development, underscoring the necessity of mental health support for breastfeeding success (Woortman et al.). For preterm infants, fortified HM supports growth and reduces complications, especially with individualized fortification methods (Ong et al.). High hydrostatic pressure (HHP) processing of donor milk preserves almost all bioactive properties, offering a viable alternative for infants unable to be breastfed (Marousez et al.).

Regarding breastfeeding safety, the COVID-19 pandemic has prompted some concerns. However, research indicates that breastfeeding remains both safe and beneficial, even when mothers test positive for SARS-CoV-2. HM from these mothers contains antibodies against the virus, providing passive immunity to infants, with proper hygiene practices ensuring safety (Smith et al.).

Finally, recent research initiatives in HM research, such as longitudinal cohort studies in China, are exploring the dynamic interplay between breast milk composition, gut microecology, and health outcomes in preterm infants (Wang et al.). Multi-omics analyses are yielding new insights into the role of HM in early development. HM banks face the dual challenge of preserving nutrient and bioactive quality, while ensuring bacteriological safety. Traditional pasteurization methods, while effective to eliminate some pathogens, may compromise certain milk components and fail to eliminate *Bacillus cereus*. Alternative methods, including HHP, UV, and non-thermal techniques, are under active investigation to address these concerns.

In conclusion, the research articles presented in this editorial unequivocally highlights the indispensable roles of HM in promoting optimal infant health and development. HM not only provides essential nutrients and bioactive compounds but also orchestrates the development of the gut microbiota, modulates immune responses, and influences gene expression through epigenetic mechanisms. Its impact on cognitive development, immune function, and long-term health outcomes is profound, offering benefits that extend well beyond infancy. The challenges associated with breastfeeding, particularly for preterm infants and mothers experiencing pain or stress, underscore the need for continued support, innovation, and research. Advances in donor milk processing and fortification, as well as ongoing investigations into the microbiome and epigenetic programming, are paving the way for improved health outcomes for all infants. Ultimately, HM remains the cornerstone of infant nutrition, immunity, and neurodevelopment, and its continued study will yield further insights into optimizing health from the earliest stages of life.

